# Glial cell line-derived neurotrophic factor (GDNF) attenuates the peripheral neuromuscular dysfunction without inhibiting the activation of spinal microglia/monocyte

**DOI:** 10.1186/s12877-018-0796-1

**Published:** 2018-05-09

**Authors:** Fei Xie, Fan Zhang, Su Min, Jingyuan Chen, Jun Yang, Xin Wang

**Affiliations:** 1 Department of Anesthesiology, the First Affiliated Hospital of Chongqing Medical University, Friendship Road 1#, Yuan Jia Gang, Chongqing, 400016 China; 2 Department of Anesthesiology, the People’s Hospital of Jianyang City, Chengdu, Sichuan China

**Keywords:** Aging, Neuromuscular dysfunction, Microglia/monocyte, Spinal cord, Nicotinic acetylcholine receptors

## Abstract

**Background:**

Peripheral neuromuscular dysfunctions were found in elderly individuals, and spinal microglia/monocyte plays an important role on this process. This study aims to test whether the glial cell line-derived neurotrophic factor (GDNF) could attenuate age-related neuromuscular dysfunction by inhibiting the activation of spinal microglia/monocyte.

**Methods:**

Male Sprague-Dawley rats were divided into an adult group and an aged group. The aged rats were intrathecally injected with normal saline (NS) and GDNF. All the rats were harvested 5 days after each injection. The muscular function was tested by compound muscle action potential, and the activation of microglia/monocyte was detected by immunofluorescence staining; cytokines were assayed by enzyme-linked immunosorbent assay; the expression level of GDNF and its known receptor GFR-α in the spinal cord, the expression level of neuregulin-1 (NRG-1) in the sciatic nerve, and the expression level of γ- and α7- ε-nicotinic acetylcholine receptors in the tibialis anterior muscle were measured by western blotting.

**Results:**

The activated microglia/monocyte was found in the aged rats compared to the adult rats. The aged rats showed a significant neuromuscular dysfunction and cytokine release as well as increased expression of γ- and α7-nAChR. The protein expression of GDNF, GFR-α, and NRG-1 in the aged rats were significantly lower than that in the adult rats. However, the exogenous injection of GDNF could alleviate the neuromuscular dysfunction but not inhibit the activation of spinal microglia/monocyte. Furthermore, the levels of GFR-α and NRG-1 also increased after GDNF treatment.

**Conclusion:**

The GDNF could attenuate the age-related peripheral neuromuscular dysfunction without inhibiting the activation of microglia/monocyte in the spinal cord.

## Background

Skeletal muscle dysfunction, which manifests as acquired muscle weakness and a reduced capacity to continue muscle contractions and other neurodegenerations, are observed in many elderly individuals [1, 2]. The demyelination of nerve has been considered the main mechanism for neuromuscular dysfunction [3]. Historically, studies have shown that the neuromuscular dysfunction was found in advanced age, and the demyelination of sciatic nerve is a factor [4]. Although the peripheral motor nerve plays an important role in muscular function, however, the central nervous system also contributes to muscular function in the elderly individuals.

The blood-spinal cord barrier (BSCB) is injured more often in the aging spinal cord than in the adult spinal cord, which was associated with increased tissue cytokine levels [5]. Microglia cell/monocyte, the resident innate immune cells in the central nervous system (CNS) that mediated neuroinflammation, are associated with many neurodegenerative disorders [6–8]. The microglia cell/monocyte exhibits greater immunoreactivity in the CNS of elderly individuals [9]. Other studies suggest that the aged microglia/monocyte cell becomes activated and leads to a status of “para-inflammation” [10–12], which is between basal homeostatic conditions and true inflammation. Even if this para-inflammation is slight, it is still detrimental to the CNS [13], and eventually aggravates peripheral motor nerve injury [14]. This indicates that peripheral nerve demyelination and muscular dysfunction may be a result of spinal cord inflammation [15].

Despite the proinflammatory effect, the microglia/monocyte exerts protective effects by secreting neurotrophic factors after being activated [16–18]. It was shown that the glial cell line-derived neurotrophic factor (GDNF) is also a very potent trophic factor for spinal motoneurons [19] and central noradrenergic neurons [20]. Therefore, the GDNF raised great expectations as a potential therapeutic agent for the treatment of neurodegenerative diseases. The GDNF has a significant effect on the remyelination of neurons following spinal cord injury [21]. Recently, the effect of GDNF on the regeneration of the peripheral nerve has since been tested in some models of nerve injury [22]. Currently, however, it is unknown whether exogenous GDNF can modulate the function of spinal microglia cell/monocyte to alleviate the peripheral neuromuscular dysfunction in the advanced age.

In previous studies, three variants of nAChRs are observed in the post-junctional synapse. Normally, ε-nicotinic acetylcholine receptors (nAChRs) are confined to the neuromuscular junctions (NMJ), which are performed the muscle contraction. However, aging leads to the re-expression of α7-nAChR and γ-nAChR in skeletal muscles and demyelination of sciatic nerve [4], which are associated with peripheral neuromuscular dysfunction.

Based on the aforementioned phenomena and conclusions, in this study, we aimed to test whether the GDNF could attenuate the age-related neuromuscular dysfunction by inhibiting the activation of spinal microglia cell/monocyte.

## Methods

### Animals

Male adult (4 months old, weight range: 220–235 g) and male aged (16 months old, weight range: 460–478 g) Sprague-Dawley rats were purchased from Chongqing Medical University (Chongqing, China). All rats received humane care according to the Animal Ethics and Use Committee of Chongqing Medical University (GB14922–2001 and GB14927–2001). All rats were housed in a SPF room with the appropriate temperature and humidity, and the normal light/dark cycle). All the procedures were reviewed and approved by the Ethical Committee of Chongqing Medical University (No.2016–46), and followed with the animal care guidelines of the National Institutes of Health.

### Group assignments

Ten male adult rats were assigned as the adult group, and 30 male aged rats were randomly divided into three groups as follows: (1) an aged group without any treatment (*n* = 10); (2) an aged group with normal saline (N.S) treated (n = 10); (3) an aged group with recombinant rat GDNF treated (Beijing Creanovo Biotechnology Co., Ltd., China, n = 10). GDNF (25 μg) or N.S was intrathecally injected into the subarachnoid space once daily injection for five days, respectively.

Intrathecal catheter implantation.

For intrathecal injection of inhibitors, the catheter implantation was performed as described previously [23, 24] with some modifications. After the animal was anesthetized by 0.5% sodium pentobarbital (65 mg/kg), a 2-cm longitudinal midline skin incision was made in the lumbar region (L4 - S1), and the intervertebral membrane between L5 and S1 was exposed. A 23-gauge needle was then used to puncture the membrane, and the PE-10 catheter was gently pushed into the L5–6 subarachnoid space at an angle of approximately 20° to 30° above the vertebral column to reach L4 at the lumbar enlargement. The inside catheter was then fixed to the muscle, and the other end was tunneled under the skin and pulled out of the cut at the neck. All incisions were closed with 4–0 silk and the neck end was sealed by heated paraffin. The rats were allowed to recover for 1 day before compound muscle action potential (CMAP) testing and intrathecal injections. The position of the catheter was checked postmortem. Animals that showed any abnormal neurological signs were excluded from experiments.

### Evaluation of neuromuscular function

On the sixth day after injections, the rats were placed in the dorsal recumbent position after being anesthetized. The right sciatic nerve was exposed at the thigh with noncompliant silk. Stimulation electrodes (BL-420S Systems, Chengdu Techman Co., LTD, China) were attached to measure nerve-mediated contraction of the tibialis anterior muscle with several parameters (intensity 2 V; duration 0.2 ms; and frequency 1 Hz). CMAP was recorded with a receiving electrode attached on the right tibialis anterior muscle [25]. The amplitude, latency time and duration of CMAP were analyzed with the BL-420S software. The motor conduction velocity was calculated as the distance of conduction/duration time. The temperature of each rat was kept at 36° to 37 °C by the heating light. A decrease of ≥20% of the lower limit of the normal CMAP amplitude could be considered as the neuromuscular dysfunction [26].

### Tissue preparation

The rats were humanely euthanatized by deep anesthesia before the collection of tissues. The lumbar enlargement, sciatic nerves and the tibialis anterior muscle specimens of rats were collected in 4% paraformaldehyde for immunofluorescence staining or frozen with liquid nitrogen for western blot analysis.

### Cytokine measurements

The lumbar enlargement samples were harvested immediately after decapitation. Samples were homogenized and were centrifuged at 2000 g for 15 min at 4 °C to separate the supernatant. The concentrations of TNF-α and IL-6 were measured by enzyme-linked immunosorbent assay (ELISA) kits (the two kits were purchased from CUSABIO BIOTECH Co., Ltd., Wuhan, China), the procedures were followed the instructions.

### Immunofluorescence staining

The deparaffinized spinal cord sections were permeabilized with 0.1% Triton X-100 and blocked with 5% normal donkey serum (Beyotime Institute of Biotechnology, Beijing, China) for 30 minutes at 25 °C. Then, the two kinds of primary antibodies including CD11b (ab8878, Abcam Ltd., dilution 1:200) and Iba-1 (ab178680, Abcam Ltd., dilution 1:200) were incubated with the spinal cord samples for 12 h at 4 °C separately. Negative controls without primary antibodies were included. After 3 washes with PBS, donkey anti-mouse Alexa-Fluor 594 (for CD11b) and donkey anti-rabbit Alexa-Fluor 488 (for Iba-1) were used as secondary fluorescent probes for 30 min at 25 °C. The sections were viewed by confocal microscopy (TCS SP2, Leica, Germany) and analyzed as individual images for CD11b and Iba-1 co-expression. The average optical density was analyzed in 10 randomly selected microscopic fields in five sections of each group at a 400-fold magnification. CD11b was quantified as the average number of positive cells per field. A negative (no antibody) control was included. All the results were recorded by researchers who were blinded to the experimental group.

### Western blot analysis

The proteins of the spinal cord, the sciatic nerves, and the skeletal muscles were prepared according to the previous studies [4]. In brief, the concentrations of proteins were determined by the BCA Protein assay kit (Beyotime, China). 50 μg proteins were separated by 10% SDS-PAGE, and then were transferred to nitrocellulose membranes. The membranes were blocked with 5% skim milk in TBS, the membranes were incubated with primary antibodies at 4 °C: GDNF (sc-13,147, Santa Cruz Biotechnology, Santa Cruz, CA; dilution: 1:600), GFR-α1 (PRS1133, Sigma-Aldrich, St. Louis, MO; dilution: 1:500), γ-nAChR (sc-13,998, Santa Cruz Biotechnology; dilution: 1:500), α7-nAChR (ab10096, Abcam; dilution: 1:800) and neuregulin-1 (NRG-1) (sc-28,916, Santa Cruz Biotechnology; dilution: 1:500). After washed with TBS containing 0.3% Tween-20, the membranes were incubated with corresponding secondary antibodies (dilution: 1:2, 000, Beyotime, China) for 1 h at 25 °C; the immunoreactive protein bands were visualized using ECL reagent (Beyotime, China), and the band intensity was performed with the Quantity One software. The relative expression levels of these proteins were expressed as values normalized to GAPDH or α-tubulin.

### Statistical analysis

All statistical analyses were performed using SPSS (version 17.0, SPSS). The results values are expressed as mean plus/minus standard deviation (SD). The data were analyzed using one-way analysis of variance to compare the within-group differences or the Student–Newman–Keuls q test to compare the between group differences. The differences in all data were considered statistically significant at a *P*-value < 0.05.

## Results

### Neuromuscular function

**Fig. 1:**
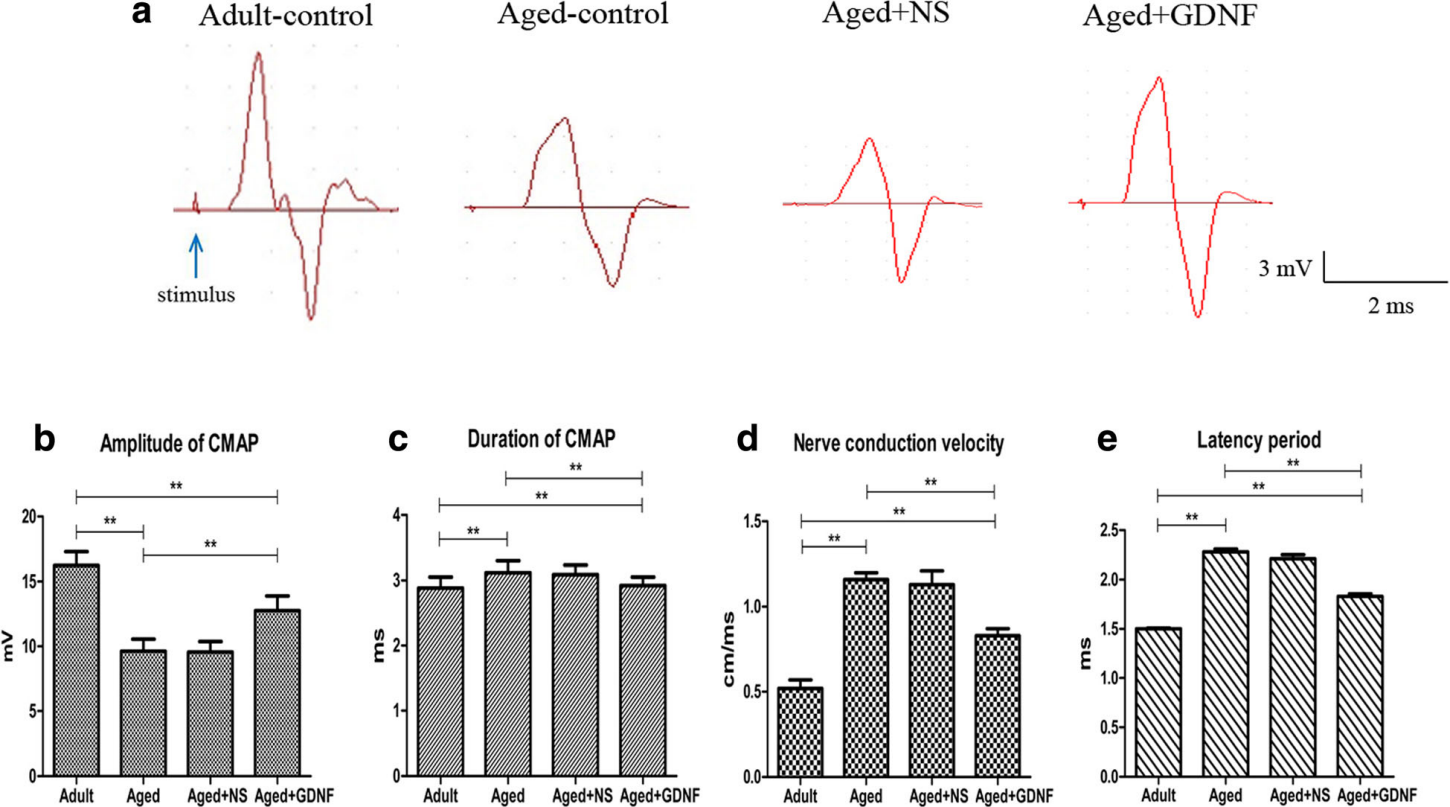
Neuromuscular function was decreased in aged rats and GDNF improved the function. **a** Samples of the CMAPs recorded in the adult group, aged group, aged + NS group and aged + GDNF group. Compared to the adult group, the amplitude was decreased in the aged group (**b**), the duration, nerve conduction velocity and latency period were increased in the aged group (**c**, **d**, **e**). The treatment of GDNF increased amplitude and decreased the duration, nerve conduction velocity and latency period. Values are shown as mean ± SD (*n* = 10, **P < 0.01)

The CMAP recorded of four groups is illustrated in Fig. 1a. In the adult group, the amplitude of CMAP was 16.23 ± 3.14 mV (Fig. 1b). However, the amplitude of CMAP decreased significantly in the aged group, (*P* < 0.01, Fig. 1b). The duration of CMAP in the aged group was significantly prolonged compared to that in the adult group (*P* < 0.01, Fig. 1c). The nerve conduction velocity was significantly prolonged in the aged group compared to that in the adult group (*P* < 0.01, Fig. 1d). The latency period was significantly prolonged in the aged group compared to that in the adult group (*P* < 0.01, Fig. 1e).

However, the amplitudes of CMAP were significantly increased in the GDNF-treated rats compared to that in the NS-treated rats (*P* < 0.05, Fig. 1b). The durations of CMAP in the NS-treated rats were significantly longer than those in the GDNF-treated rats (*P* < 0.05, Fig. 1c). The nerve conduction velocity significantly decreased in the GDNF-treated rats compared to the NS-treated rats (*P* < 0.05, Fig. 1d). Although the latency period was significantly increased in the NS-treated rats (*P* < 0.05, Fig. 1e), it was significantly decreased in the GDNF-treated rats (*P* < 0.05).

### Cytokine plasma levels

**Fig. 2:**
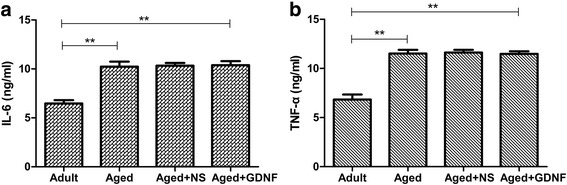
Cytokines (TNF-α and IL-6) in the spinal cord for the four groups are shown (**a** and **b**). Compared to the adult group, the cytokines were relatively increased in the aged group. The cytokines were still at high level after GDNF treatment compared to the aged group. Values are shown as mean ± SD (*n* = 10, **P < 0.01)

The concentrations of cytokine (TNF-α and IL-6) are presented in Fig. 2a and b. The cytokine level in the spinal cord, which was determined by ELISA, was low in the adult group. Compared to the adult group, the cytokine level was relatively increased in the aged group (*P* < 0.01). However, the level of cytokines was still high in the GDNF-treated group compared to that in the aged group (*P* > 0.05).

### Immunofluorescence staining

**Fig. 3:**
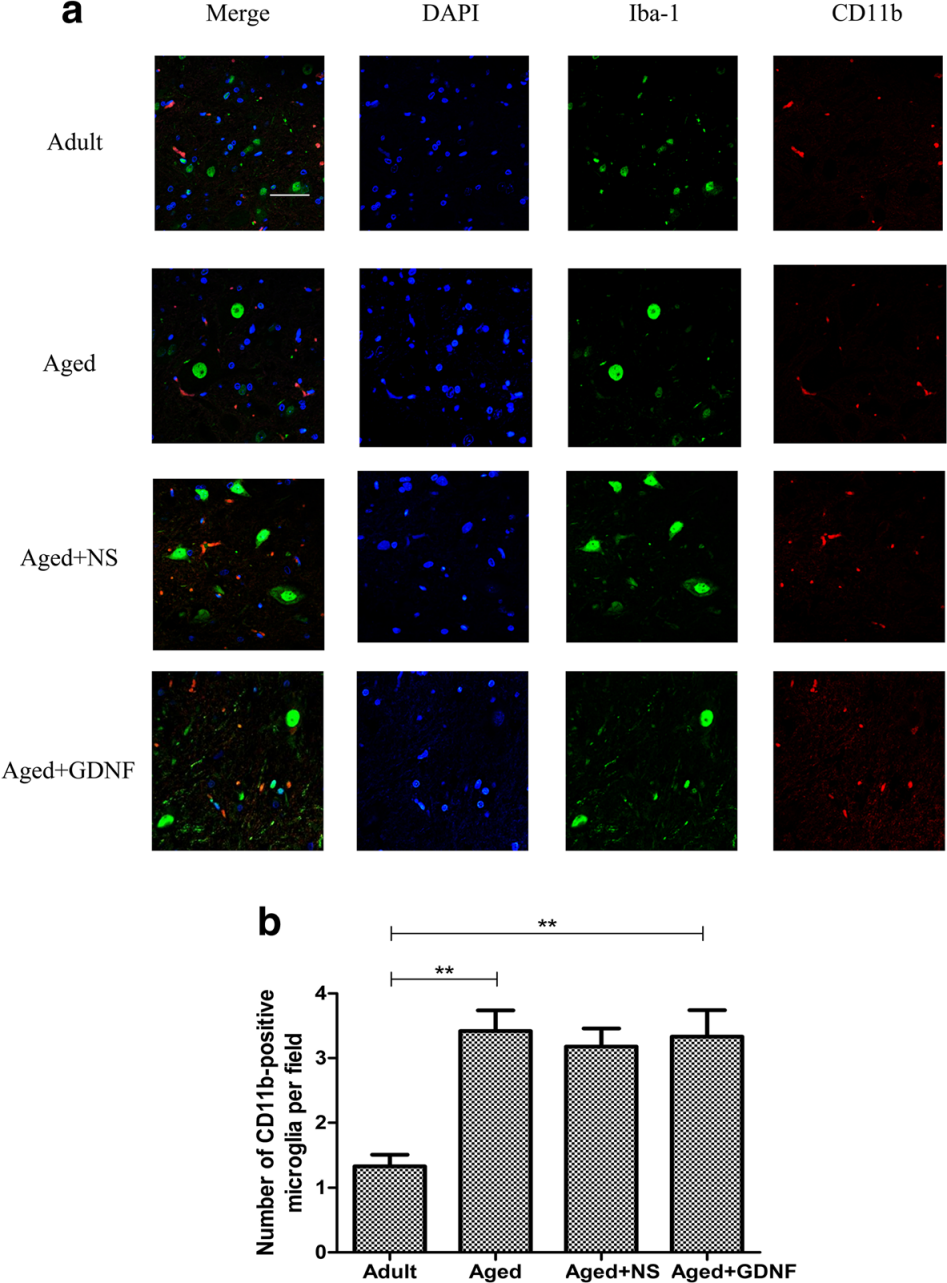
The activation of Iba-1-positive cells co-labeled with CD11b were found in aged rats. **a** Representative Iba-1-positive cells co-labeled with CD11b in the spinal tissue. **b** Quantification showed that the aged rats had a relatively large number of Iba-1-positive cells co-labeled with CD11b in the spinal tissue than in the adult rats. However, the number of Iba-1-positive cells co-labeled with CD11b had no change in the GDNF-treated rats. The total number of Iba-1-positive cells co-labeled with CD11b was expressed as the mean number per field of view. Values are shown as mean ± SD (*n* = 10, *P < 0.05, **P < 0.01). Bar = 40 μm

The immunofluorescence staining results of CD11b-positive microglia/monocyte in the 4 groups are shown in Fig. 3a. Few or no CD11b-positive microglia cells/monocytes were detected in the adult group. However, an increasing number of activated CD11b-positive microglia cells/monocytes were observed in the aged group. These activated microglia cells/monocytes exhibited relatively robust CD11b immunoreactivity compared to the adult group (*P* < 0.01, Fig. 3b). GDNF could not inhibit the increase in CD11b-positive microglia cells/monocytes of aged rats (*P* < 0.05, Fig. 3b), and microglia cells/monocytes still exhibited reactive morphology.

### Western blot results

**Fig. 4:**
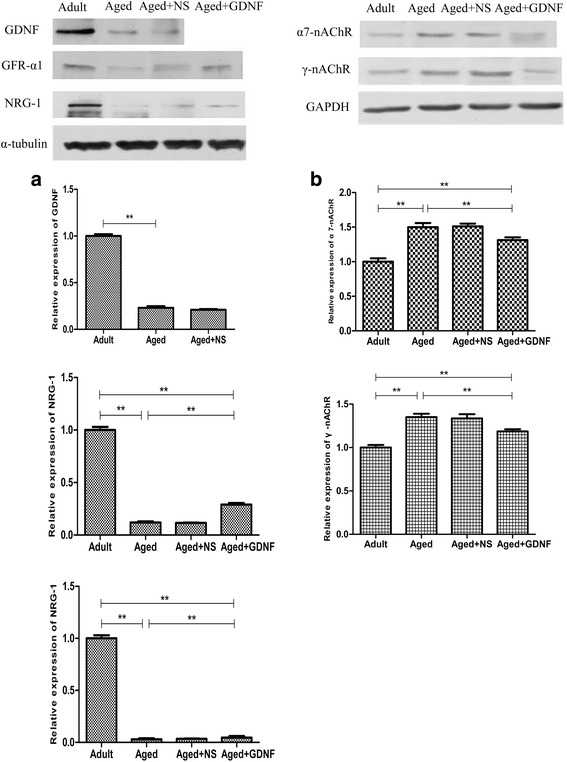
Compared to the adult rats, the protein expression levels of GDNF, GFR-α, and NRG-1 were significantly decreased in the aged group (**a**), whereas the protein expression levels of α7- and γ-nAChR were increased (**b**). The protein expression level of α7- and γ-nAChR was significantly increased in the aged rats. GDNF treatment decreased the protein expression of α7- and γ-nAChR but increased that of GFR-α and NRG-1 5 days postinjection (**a** and **b**). Values are shown as mean ± SD (*n* = 10, **P < 0.01)

Western blotting showed that the protein expression of NRG-1, GDNF, and GFR-α1 were significantly decreased in the aged group (*P* < 0.01, Fig. 4a). On the contrary, compared to the adult group, the protein expression levels of α7- and γ-nAChR were increased in the aged group compared to those in the adult group (*P* < 0.01, Fig. 4b).

The protein expressions of α7- and γ-nAChR were decreased after treatment with GDNF (*P* < 0.01, Fig. 4a). In contrast, the protein expression levels of NRG-1 and GFR-α1 were significantly increased in the GDNF-treated group compared to those in the NS-treated group (*P* < 0.01, Fig. 4b).

## Discussion

In this study, we showed that the GDNF attenuates the age-related neuromuscular dysfunction without inhibiting the activation of spinal microglia/monocyte. Our results showed that the CD11b-positive microglia/monocytes were activated and released a few cytokines in the aged spinal cord. Intrathecal injection of GDNF neither decreased the inflammatory response nor deactivated the microglia/monocytes in the aged spinal cord. According to our results, the insufficient level of GDNF could be one of the factors that induced neuromuscular dysfunction in aged rats.

The activation of microglia/monocytes results in chronic inflammation, a dysregulated or uncontrolled response to injury, and poorer recovery in advanced age [27]. The elevated basal cytokine level in the spinal cord may contribute to the higher activated state observed in these microglia/monocyte populations, especially with age, leading to a pro-inflammatory feedback loop that disrupts normal maintenance functions [5].

Previous studies also suggested that normal healthy aging was accompanied by neuroinflammation, which enhanced susceptibility to neurodegeneration [28, 29]. For example, multiple inflammatory markers increase with age in various brain and spinal cord regions of healthy rats, mice, and primates [5, 29–32]. In other words, the ability of senescent CNS to react to deleterious neurotoxic responses is decreased following injury, infection, or stress [33]. Furthermore, neuroimmune profiles of healthy aged CNS suggest that activation of microglia/monocytes [34] may contribute to neuromuscular disorders.

Although inflammation is associated with neuromuscular function in advanced age, the lack of neurotrophy could be another factor that could induce the dysfunction. GDNF could regulate the development and the differentiation of cells and neurons [35]. In addition, during some pathophysiological conditions, GDNF is upregulated in microglia/macrophages [36, 37]. Some studies showed that GDNF was secreted after the activation of microglia cells/monocytes in the CNS [38, 39]. However, in this study, we found that even if there was slight inflammation in the aged spinal cord, the neuromuscular dysfunction was significantly alleviated by exogenous injection of GDNF. Therefore, the insufficient level of GDNF in the aged spinal cord may be a reason for neuromuscular dysfunction.

Our previous studies demonstrated that aging induced upregulation of γ- and α7- nAChR with downregulation of NRG-1 [4]. The expression of NRG-1 on the sciatic nerve may reflect the thickness of the myelin sheath [40]. In addition, NRG-1 had a regulatory role on the synthesis, expression, and stability of postsynaptic nAChRs through the MAP kinase signaling pathway, which were associated with the activation and expression of nAChRs [41, 42].

In this study, we found that the expression of GDNF was decreased in aged rats. Exogenous injection of GDNF could upregulate the expression of NRG-1 in the sciatic nerve and downregulate the expression of γ- and α7- nAChR in skeletal muscles. The signaling mechanisms between GDNF and NRG-1 need more studies. Although the precise consequences of the upregulation of microglia/monocyte-derived GDNF in CNS inflammation have not been elucidated, enhancing the level of GDNF may contribute to a future therapeutic strategy for the treatment of inflammatory demyelinating diseases.

The spinal microglia was activated in advanced age, which was a factor of the neuroinflammation in the spinal cord. Previous studies had found that rat microglia express both Gfr-α1 and c-Ret, and our data supported these results. Interestingly, the GDNF-induced activation of c-Ret and subsequent phosphorylation of Erk1/2 has been previously reported in rat microglia [43, 44]. In this study, we demonstrated that GDNF was not able to inhibit aging-induced microglia activation, according to the expression and release of IL-6 and TNF-α. These results indicate GDNF might also have c-Ret-independent functions in rat microglia.

Our study also had some limitations. The administration of GDNF is a short term treatment rather than a long term treatment. However, based on previous studies [45], we found that intrathecal injection of GDNF for 3 consecutive days could attenuate the mechanical allodynia and thermal hyperalgesia at 24 h after the first GDNF injection, and the analgesic effects continued for at least 3 days after the last injection. Thus, even though the microglia/monocytes had not been inactivated by GDNF, we proposed that the GDNF had enough effect after the last injection.

It had been reported that the microglia of female mice may express higher levels of cytokines [46]. These differences are associated with the secretion of testosterone surge in male mice after birth. Previous studies also proved that androgens can reduce the expression of proinflammatory cytokines in macrophages [46–48]. In addition, although there are no obvious differences in microglial gene expression between male and female, the function of microglial may be associated with sex differences. However, the male rats were examined in this study, so we should still consider the different microglial properties.

This study was performed using rat tissue and although the data had not suggested a regulatory potential of GDNF to downregulate microglia activation, it is still an open issue whether GDNF is able to directly inhibit aging-induced microglia activation.

## Conclusions

Our findings demonstrated a vital role for GDNF in spinal microglia/monocytes in aged rat. The lack of GDNF in the aged spinal cord is associated with neuromuscular dysfunction, and exogenous injection of GDNF in the spinal cord improved the function recovery; however, GDNF could not inhibit the activation of microglia/monocytes.

## Abbreviations

ANOVA: Analysis of variance
CMAP: Compound muscle action potential
CNS: Central nervous system
DAPI: 4′,6-diamidino-2-phenylindole
ELISA: Enzyme-linked immunosorbent assay
GAPDH: Glyceraldehyde-3-phosphate dehydrogenase
GDNF: Glial cell line-derived neurotrophic factor
MCV: Motor conduction velocity
MyD88: Myeloid differentiation factor 88
NF-κB: Nuclear factor-kappa B
NRG-1: Neuregulin-1
NS: Normal saline
PBS: Phosphate-buffered saline
PVDF: Polyvinylidene fluoride
s nAChR: nicotinic acetylcholine receptor
SD rats: Sprague-Dawley rats
SDS-PAGE: Sodium dodecyl sulfate polyacrylamidegel electrophoresis
SNK: Student–newman–keuls
TNF-α: Tumor necrosis factor alpha
